# Loose Intraperitoneal Spiral Tack Causing Small Bowel Obstruction Following Laparoscopic Intraperitoneal Onlay Mesh Repair of Ventral Incisional Hernia: A Case Report

**DOI:** 10.7759/cureus.64424

**Published:** 2024-07-12

**Authors:** Ajay N Mistry, Mamoon Solkar, Mostafa Abdel-Halim

**Affiliations:** 1 General Surgery, Tameside General Hospital, Ashton-Under-Lyne, GBR

**Keywords:** incisional ventral hernia, bowel obstruction, surgical tack migration, ipom, intraperitoneal onlay mesh

## Abstract

This case report discusses a rare complication of small bowel obstruction occurring in the early course following Intraperitoneal Onlay Mesh (IPOM) repair for an incisional hernia. The bowel obstruction, which failed to respond to conservative measures, was caused by band adhesions resulting from the presence of a loose intraperitoneal migrated surgical tack. This was successfully managed laparoscopically resulting in complete recovery. We present the clinical and radiological findings and review the relevant literature in this area.

## Introduction

Incisional hernia is a common complication following abdominal surgery and is usually associated with significant morbidity and symptom burden for the patient [1,2]. Ongoing symptoms can lead to significant alterations to the patient’s lifestyle and employment. Moreover, a significant proportion of patients present with acute irreducibility and strangulation requiring emergency surgery [3].

Surgical repair usually involves either an open approach in which the mesh is placed within the retro-muscular or preperitoneal planes, or a laparoscopic approach in which the mesh is placed in the peritoneal cavity. More recently, robotic surgery has introduced a minimally invasive approach that enables the surgeon to place the mesh as would be achievable in an open approach [4,5].

Laparoscopic intraperitoneal on-lay mesh (IPOM) repair is a common operation employed for incisional hernia repair in which a mesh is introduced into the peritoneal cavity to cover the hernial defect and is secured into position with the use of tacks [6].

Whilst considered safe and effective, complications can arise postoperatively. These are described as either minor complications, including hematoma and seroma formation, or major complications including bowel-related complications, intra-abdominal adhesions, and mesh infection [7,8].

We present the case of a unique complication from an IPOM procedure in which acute small bowel obstruction occurred six weeks post-operatively due to migration of a surgical tack resulting in an adhesional band and subsequent ileal obstruction.

## Case presentation

A 78-year-old male presented with symptoms indicative of small bowel obstruction, including abdominal pain, distension, and vomiting, six weeks after undergoing IPOM repair for an incisional umbilical hernia. The 6cm hernial defect was repaired with a 20x15cm composite mesh to achieve adequate fascial overlap. Mesh fixation was performed using Medtronic ProTackTM fixation device (helical non-absorbable tacks). At the index hernia repair operation, it was reported that two concentric circles of tacks were placed achieving adequate and secure contact of the mesh to the abdominal wall, and there was no report of loose tacks by the operating surgeon. A CT scan of the abdomen and pelvis showed adhesional small bowel obstruction. Conservative management with nasogastric tube decompression and intravenous fluid therapy was therefore initiated. However, the patient's condition did not improve, and high output from the nasogastric tube persisted. Moreover, gastrografin follow-through studies indicated no passage of contrast beyond the distal small bowel.

**Figure 1:**
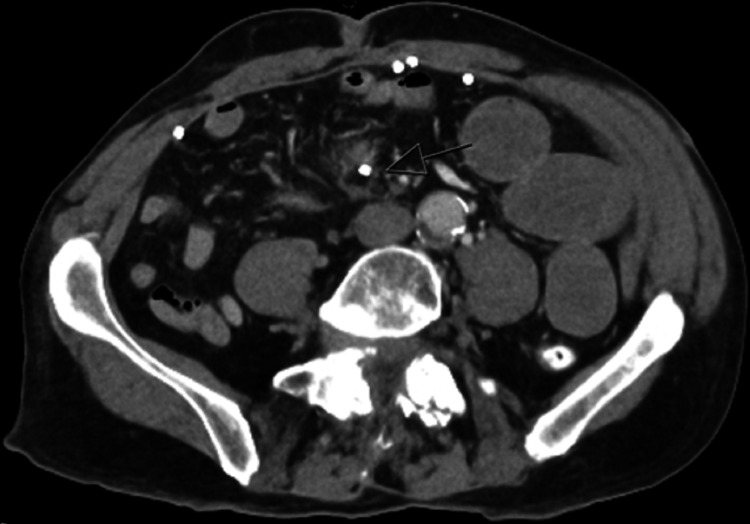
Axial CT image with an arrow showing the loose tack at the transitional point.

**Figure 2:**
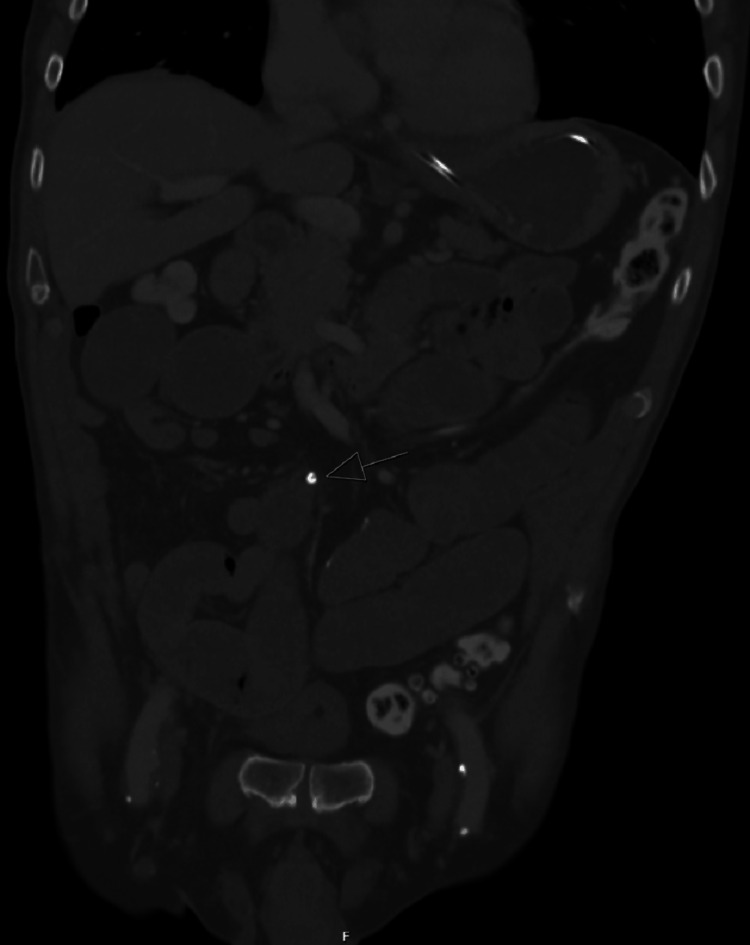
Coronal CT (bone view) image with an arrow showing the loose tack at the transitional point.

The failure of conservative treatment after 3 days prompted further assessment with a repeat CT scan of the abdomen and pelvis. This revealed significant dilatation of the small bowel loops, with an abrupt transition point identified at the level of the ileum. Notably, a rounded hyperdense structure measuring up to 7mm, suspected to be a migrated surgical tack, was observed at the ileal transition point, surrounded by inflammatory strandings (Figure 1, 2).

**Figure 3:**
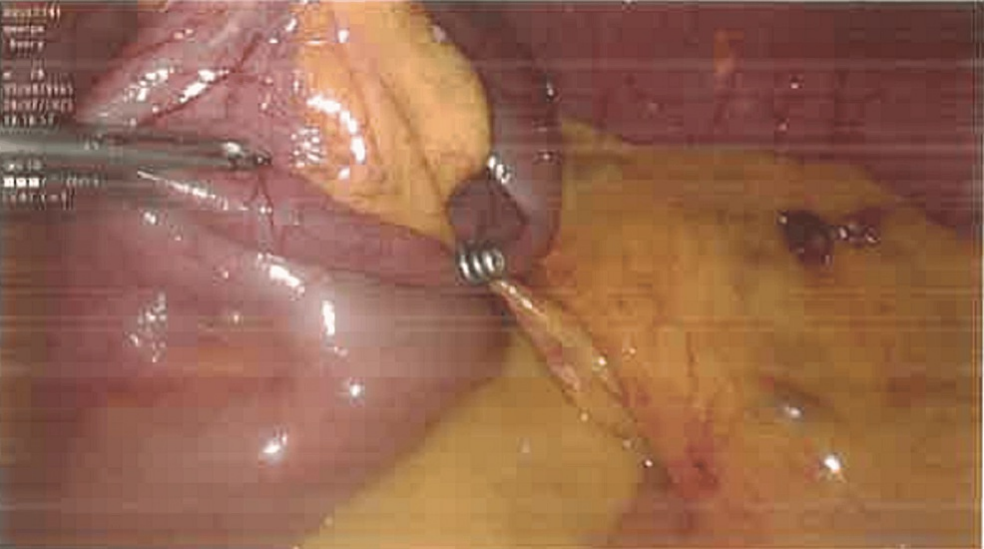
Operative photo demonstrating the loose tack attached to an ileal loop and the obstructing omental band.

**Figure 4:**
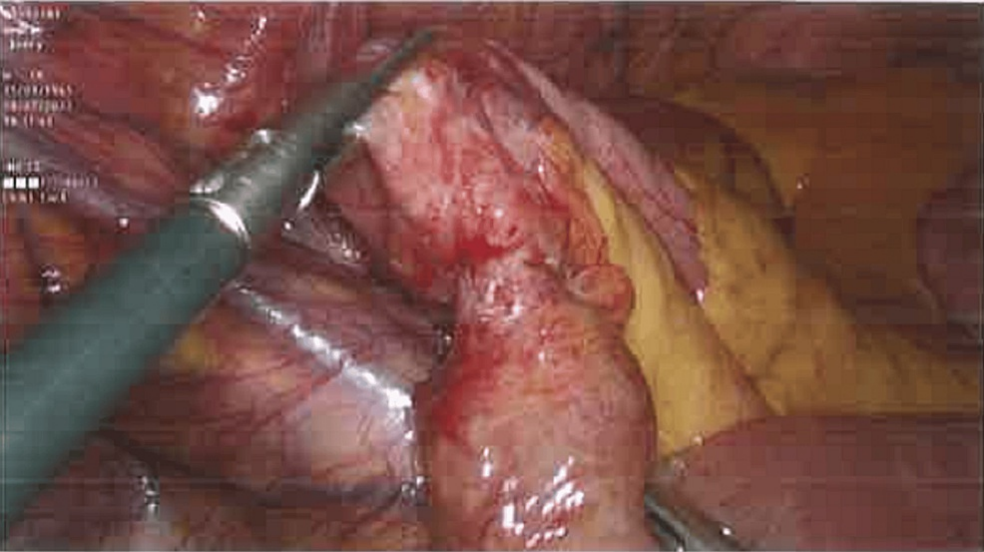
Operative photo of the small bowel constriction ring following the removal of the tack and excision of the obstructing band.

Emergency laparoscopy was subsequently performed revealing four migrated surgical tacks found loose within the peritoneal cavity, with one of them causing an obstructing band adhesion around a distal ileal loop (Figure 3, 4). The other three were causing non-obstructing omental adhesions. Laparoscopic complete adhesiolysis with division of the obstructed band adhesion and extraction of the loose tacks from within the peritoneal cavity and the mesentery was carried out. The patient subsequently made an uneventful recovery and was discharged home on the third postoperative day.

## Discussion

In this case report, we discuss a rare complication whereby tack migration has resulted in the development of an obstructive adhesional band and subsequent small bowel obstruction.

The advent of laparoscopic techniques, including the IPOM repair, has significantly improved outcomes in incisional hernia patients [9]. However, a limited but growing body of evidence highlights the increasing recognition of tack-related complications, including adhesional bowel obstruction, which poses a substantial risk and necessitates prompt intervention [10,11].

The migration of surgical tacks within the peritoneal cavity can lead to serious complications, including bowel obstruction, as demonstrated in this case. Tacks may also migrate through the bowel wall, causing luminal obstruction, inflammation, and potential perforation. To mitigate such risks, it is imperative for surgeons to exercise meticulous care during tack deployment. One potential solution is to adopt a standardized protocol that involves systematic checking and accounting for each tack following deployment. This would not only ensure the secure fixation of the mesh but also reduce the likelihood of migration-related complications [12,13]. Other technical considerations in this regard include the use of glue instead of tacks for mesh fixation or the use of a self-fixating mesh [14].

Our case report represents a rare incident in which tacks were migrated loose into the peritoneal cavity leading to the formation of obstructing band adhesions. Our literature review did not reveal any similar reports. Increased awareness of such tack-related complications amongst radiologists is warranted as an accurate characterization of the CT findings will influence the surgical management decision. In our case, the interpretation of the initial CT scan was of postoperative adhesions. The presence of migratory tacks was missed, and the presence of the radio-opaque migrated tack was noted only on subsequent repeat imaging.

Alternative incisional hernia repair techniques, such as the Rives-Stoppa procedure involve placing the mesh in a retro-rectus plane through an open surgical approach, avoiding the risks associated with the placement of an intraperitoneal mesh and tacks [15]. However, other advantages and disadvantages of each approach, including the implications of open versus laparoscopic approaches, are all to be considered when decisions are made for individual cases. Moreover, factors such as the hernia size, patient anatomy, and overall fitness will have an impact on the surgical approach adopted in each patient [16].

Evidence on absorbable versus non-absorbable tacks suggests no difference in outcomes, namely recurrence, hematoma risk, and seroma risk [17]. It is difficult to predict if a complication such as the one we have reported would have been avoided had the operating surgeon used absorbable tacks. However, as the evidence suggests that hernia outcomes are comparable, we believe that it would be favorable to use absorbable tacks or alternative techniques of mesh fixation.

## Conclusions

The increasing recognition of complications related to tack migration following IPOM procedures, including adhesional bowel obstruction, underscores the importance of vigilant surgical techniques. Adopting careful tack deployment protocols and considering alternative mesh fixation techniques can contribute significantly to reducing the incidence of tack migration-related complications and enhance the overall safety and efficacy associated with this procedure.
